# Transcriptome analysis of head kidney in grass carp and discovery of immune-related genes

**DOI:** 10.1186/1746-6148-8-108

**Published:** 2012-07-09

**Authors:** Jin Chen, Cai Li, Rong Huang, Fukuan Du, Lanjie Liao, Zuoyan Zhu, Yaping Wang

**Affiliations:** 1 State Key Laboratory of Freshwater Ecology and Biotechnology, Institute of Hydrobiology, Chinese Academy of Sciences, Wuhan, 430072, China; 2 Graduate School of Chinese Academy of Sciences, Beijing, 100039, China

**Keywords:** Grass carp, Head kidney, cDNA, EST, Immune-related gene

## Abstract

**Background:**

Grass carp (*Ctenopharyngodon idella*) is one of the most economically important freshwater fish, but its production is often affected by diseases that cause serious economic losses. To date, no good breeding varieties have been obtained using the oriented cultivation technique. The ability to identify disease resistance genes in grass carp is important to cultivate disease-resistant varieties of grass carp.

**Results:**

In this study, we constructed a non-normalized cDNA library of head kidney in grass carp, and, after clustering and assembly, we obtained 3,027 high-quality unigenes. Solexa sequencing was used to generate sequence tags from the transcriptomes of the head kidney in grass carp before and after grass carp reovirus (GCRV) infection. After processing, we obtained 22,144 tags that were differentially expressed by more than 2-fold between the uninfected and infected groups. 679 of the differentially expressed tags (3.1%) mapped to 483 of the unigenes (16.0%). The up-regulated and down-regulated unigenes were annotated using gene ontology terms; 16 were annotated as immune-related and 42 were of unknown function having no matches to any of the sequences in the databases that were used in the similarity searches. Semi-quantitative RT-PCR revealed four unknown unigenes that showed significant responses to the viral infection. Based on domain structure predictions, one of these sequences was found to encode a protein that contained two transmembrane domains and, therefore, may be a transmembrane protein. Here, we proposed that this novel unigene may encode a virus receptor or a protein that mediates the immune signalling pathway at the cell surface.

**Conclusion:**

This study enriches the molecular basis data of grass carp and further confirms that, based on fish tissue-specific EST databases, transcriptome analysis is an effective route to discover novel functional genes.

## Background

Grass carp (*Ctenopharyngodon idella*) is one of the most important freshwater fish, with fast growth, low cost of breeding, and delicious meat. It is widely distributed in China's major river systems. Grass carp is a farmed species that is easily affected by diseases induced by viruses and bacteria; this can cause tremendous economic losses. To date, no excellent breeding varieties have been obtained by the oriented cultivation technique. Because of the long breeding cycle (4–5 years), a hybrid breeding strategy is not feasible. Further, because of the lack of understanding of the genetic background of grass carp, no molecular breeding technology has been applied. The discovery of economically important trait-related genes and their functional study may help to establish a molecular breeding technology system in the fish.

ESTs (expressed sequence tags) are partial cDNA sequences obtained after sequencing the ends of random cDNA clones. ESTs were first used in 1991 as an effective new method to discover human genes. Using EST sequences, unknown genomes could be explored at a relatively low cost [1]. With the development of DNA sequencing technology, the cost of sequencing is becoming lower, and the application of large-scale EST sequencing combined with bioinformatics tools for analyzing data is being widely used in different species to find novel genes, for genome annotation, for the identification of gene structure and expression, and in the development of type I molecular markers [2]. In fish, large scale EST sequencing was used in channel catfish (*Ictalurus punctatus*) [3], common carp (*Cyprinus carpio*) [4], and zebrafish (*Danio rerio*) [5].

In recent years, high-throughput data analysis methods have gradually improved and the genomes of many kinds of fishes have been studied. The fishes that have been studied include zebrafish [6] and fugu [7], as model organisms, and the commercial fishes such as Atlantic salmon [8], sea bass [9,10], rainbow trout [11], Atlantic halibut [12], bluefin tuna [13], turbot [14,15], and Senegal sole fish [16]. In contrast, the molecular biology of grass carp is relatively unknown; currently, there are only 6,915 grass carp ESTs in NCBI’s dbEST database. Most functional genomic research on economically important fish is focused mainly on the development of molecular markers, genetic map construction and gene interval mapping, and other basic data accumulation. Research into gene function and its application to breeding is still in the initial stages.

Head kidney is an important immune organ in teleost fish; its role is equivalent to mammalian bone marrow [17]. Head kidney contains a large number of T and B lymphocytes, macrophages and granulocytes that are the basis upon which specific and non-specific immunity is acquired.

In this study, we constructed a non-normalized cDNA library for the head kidney of grass carp and obtained 3,027 unigenes including 221 genes of unknown function. We compared the head kidney expression profiles of grass carp infected with grass carp reovirus (GCRV) with normal controls and obtained 22,144 differential expressed tags. Based on a comparison of the differential expressed tags and potential genes with unknown function in the cDNA library, and by identifying gene expression response to GCRV and predicting protein structure, we discovered a novel immune-related gene. This study provides a method for the discovery of novel genes, and reveals the function and the network regulation mechanism of immune-related genes. The results provide a theoretical foundation for molecular design breeding in grass carp.

## Methods

### RNA extraction and construction of the cDNA library

Total RNA was extracted from the head kidney of healthy adult grass carp using Trizol reagent (Invitrogen, Carlsbad, CA, USA). The mRNA was isolated using the Oligotex mRNA Kit (QIAGEN, Hilden, Germany). Full length cDNA was synthesized by the Creator^TM^ SMART^TM^ cDNA Library Construction Kit (Clontech, CA, USA) following the method described previously [18]. cDNA segments longer than 1 kb were isolated by electrophoresis, then ligated into pDNR-LIB vector (Clontech) and used to transform competent *E. coli* DH5α cells. After growing the colony for 12 hours on an LB plate containing chloramphenicol, the cDNA library was constructed by selecting mono-clones from the 96-well plate. Ethical approval for the work was obtained from Expert Committee of Biomedical Ethics, Institute of Hydrobiology of the Chinese Academy of Sciences. The Reference number obtained was Y12202-1-303.

### DNA sequencing and processing of the EST sequences

10,464 clones were randomly selected from 109 96-well plates. After extracting the recombinant plasmids, 5’ terminal sequencing was performed using the T7 universal primer (T7: 5′-TAATACGACTCACTATAGGG-3′; Tm = 53.2 °C).

An optimal peak chart was obtained by processing the raw sequence data with basecalling. Next, FASTA format sequences (raw ESTs) were obtained by processing the optimal peak chart using the Phrap program [19] with the Q20 standard. We used crossmatch (Smith and Green, unpublished observations) to remove the pDNR-LIB vector sequences and after excluding EST sequences that were less than 100 bp long, we obtained a cleaned EST data set. Clustering of the cleaned ESTs was performed using UIcluster [20]. The UIcluster sequences were assembled using the Phrap program to build a unigene data set for the ESTs from the head kidney of grass carp.

### BLAST searches, GO functional classification and KEGG pathway analysis

We used the NCBI BLAST server [21] to identify sequences that were similar to the sequences in the NCBI nucleotide sequence database (Nt), the protein sequence database (Nr) [22] and the Swissprot database [23] using BLASTN, and BLASTX [24]. Using the EST sequence with the highest homology as a guide, we set the threshold E-value to E < 1e-6.

We used the BLASTX search results from the Swissprot database and the Blast2GO tool [25] to assign GO functional classification to the unigene sequences. Blast2GO parameters were set as follows: E-Value-Hit-Filter < 1e-6; annotation cutOff = 55; other parameters remained at the default values.

KAAS [26] was used to assign the unigene ESTs to pathways based on KEGG Orthology (KO) [27]. Unigenes were mapped to the corresponding KEGG pathways using the comparison method of bi-directional best hit.

### GCRV infection of grass carp and preparation of RNA sample

The GCRV-873 strain was provided by the Gaobo biotechnology company (Wuhan, China). One-year-old grass carp with an average weight of 180–210 g were intraperitoneally injected with 150–200 μL GCRV, a dosage of approximately 10^6^ TCID_50_ kg^-1^ body weight. The injected grass carp were raised in clean tanks at 28°C. Three infected grass carp with typical hemorrhage symptoms (infected group, n = 3) and three uninfected grass carp (healthy control group, n = 3) were selected at 5d after infection for further study. Total RNA was extracted from the head kidney of both groups using Trizol reagent. cDNA was obtained after reverse transcription and used for Solexa sequencing.

Three-month-old grass carp with an average weight of 30–60 g were intraperitoneally injected with 50–80 μL GCRV, a dosage of approximately 10^6^ TCID_50_ kg^-1^ body weight; fish in the control group were injected with same amount of saline. The grass carp were raised in clean tanks at 28°C. At 1d, 2d, 3d, 4d, 5d after infection ten GCRV-infected carp were selected for further study (n = 10). Ten uninfected fish were selected from the control group at 0d (n = 10). The whole fish was immediately used for RNA isolation. cDNA was obtained after reverse transcription and used for the detection of gene expression.

### Solexa sequencing and expression profile analysis

The *Nla*III and *Mme*I digestion method [28] was used to build a 21-bp cDNA tag library of the two groups (one-year-old), the control group and the GCRV-infected group. The tags in the two libraries end with different Illumina adapter sequences. The raw sequencing read length was 35 bp. The Solexa sequencing was performed by the Beijing Genomics Institute (BGI, Shenzhen, China).

The raw sequence data was processed through basecalling, the adapter and low quality sequences were removed, and cleaned 21-bp tags were obtained. We converted the cleaned tag number into the standard (relative) number of transcripts per million (TPM), and calculated the logarithm of TPM for each of the cleaned tags from the control and GCRV-infected groups. We used a dual limit of P <0.01 and FPR (false positive rate) <0.01, to find cleaned tags with log2Ratio ≥ 1 or log2Ratio ≤ −1 [29]. The selected tags have differential expression levels of more than 2-fold in both groups. We then compared the differential expressed tags with the unigenes from the cDNA library using SeqMap [30]; mismatch was set to 0, and sense and antisense strands were considered in the mapping.

### Semi-quantitative RT-PCR and RACE cloning

Total RNA was used to synthesize the first strand cDNA. Upstream and downstream primers (Table 1) were designed based on the unigene sequences. β-actin (primers, β-actin-F and β-actin-R) was used as the internal reference. PCR and electrophoresis was used to detect the change of expression level.

**Table 1:** Primers used for semi-quantitative RT-PCR and RACE

**Primer**	**Sequence (5′ to 3′)**	**Application**
291-F1	ATGTGGGTGATAGTTGGTTTACAAT	Expression study
291-R1	GTAATTTCAGAAGCACAGTTGAGAG	Expression study
357-F1	CTATCGCATGATTGCCTACTCAGACT	Expression study
357-R1	ACAACATTTTCCATCTCAATCTCAG	Expression study
788-F1	GGTCTTAACGGAGAGAAGTGCGA	Expression study
788-R1	GACTCTTCCGGCACGTAACT	Expression study
153-F1	CCAGCATCACAGTGTTCAGGCAG	Expression study
153-R1	AGTGTGTAGTTGTGTTCACCCTCC	Expression study
β-actin-F	CAGATCATGTTTGAGACC	Expression study
β-actin-R	ATTGCCAATGGTGATGAC	Expression study
291-F2	CTCTCAACTGTGCTTCTGAAATTAC	3’ RACE PCR
291-R2	ATTGTAAACCAACTATCACCCACAT	5’ RACE PCR
357-F2	GGTATGATTATGACTAAAGCAGGAC	3’ RACE PCR
357-R2	GTCCTGCTTTAGTCATAATCATACC	5’ RACE PCR
788-F2	AGTTACGTGCCGGAAGAGTC	3’ RACE PCR
788-R2	TCGCACTTCTCTCCGTTAAGAC	5’ RACE PCR
153-F2	GGAGGGTGAACACAACTACACACT	3’ RACE PCR
153-R2	CTGCCTGAACACTGTGATGCTGG	5’ RACE PCR

3' and 5' RACE was performed using the BD SMART RACE cDNA Amplification Kit (Clontech) according to the manufacturer’s instructions. Upstream and downstream primers used in the 3' and 5' RACE were designed based on the EST sequences (Table 1). Full length cDNA sequences of each gene were assembled using the 3' and 5' terminal sequences.

## Results

### Head kidney cDNA library of grass carp

The storage capacity of the original library was 6 × 10^5^, in the form of the *E. coli* DH5α cells that were stored on the 532 96-well plates in a total of 51,072 clones. One hundred randomly selected clones were used for further study. The PCR test results showed that the size of inserts was between 1–3 kilobases, the library reorganization was 97.85% and the no-load rate was 2.15%.

### EST sequence analysis

**Figure 1:**
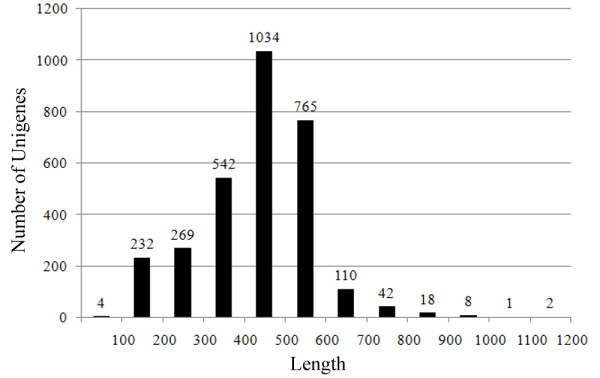
**Length distribution of the assembled EST unigenes.** The abscissa indicates the length of the unigenes, the ordinate indicates the number of unigenes.

10,464 EST clones were sequenced, and 10,282 FASTA sequences (raw ESTs) with an average read length of 470 bp were obtained. After removing the vector and sequences less than 100 bp long, 7,918 cleaned ESTs (accession no. JK847435-JK855352) were obtained. After clustering and assembly, we obtained 3,027 unigene EST sequences, 802 (26.5%) of which were contigs and 2,225 (73.5%) of which were singletons; the library redundancy was 61.78%. Most genes in the library exhibited low-level expression, only a small number of genes exhibited high-abundance expression. The number of low expression unigenes, the singletons, was 2,225 (73.5%); the number of medium expression unigenes, those containing 2–5 ESTs was 641 (21.2%); and the number of high expression unigenes, those that contained six or more ESTs, was 161 (5.3%). Only 23 unigenes contained more than 20 ESTs. The average length of the unigenes was 431 bp and 77.33% of the unigenes were 300–500 bp long (Figure 1).

### BLAST searches and GO functional classification

The 3,027 unigenes were used as queries in BLAST searches of the NCBI nucleotide and protein sequence databases and the Swissprot database. 2,713 unigenes (89.6%) matched sequences in the nucleotide sequence database, 2,162 unigenes (71.4%) matched sequences in protein sequence database and 1,845 unigenes (61.0%) matched sequences in the Swissprot database. In all, 2,806 unigenes (92.7%) matched sequences in at least one of the three databases; the remaining 221 unigenes (7.3%) were not found (E-value <1e-6) in any of the three databases and may be novel gene sequences.

Using the gene ontology (GO) classification, we successfully assigned functional annotations to 1,323 of the unigene sequences. In the GO biological process ontology, three terms accounted for the largest proportion of unigenes, they were cellular process, metabolic process and biological regulation; in the GO molecular function ontology, the three most commonly occurring terms were binding, catalytic activity and structural molecule activity; and in the GO cellular component ontology, cell, cell part and organelle were the terms that occurred most frequently (Table 2). Of the 1,323 GO-annotated unigenes, 53 were immune system process-related genes (Table 3), 4 were response to virus, and 9 were response to bacterium process-related genes (Tables 4 and 5). Some unigenes were assigned multiple functions. Not all of the unigenes could be mapped to the lower level GO terms.

**Table 2:** GO functional classification of the unigene data set

	**GO term**	**Number of unigenes**	**%**
**Biological Process**	cellular process	899	68.0
	metabolic process	672	50.8
	biological regulation	379	28.6
	regulation of biological process	356	26.9
	localization	250	18.9
	establishment of localization	232	17.5
	developmental process	135	10.2
	response to stimulus	126	9.5
	multicellular organismal process	100	7.6
	positive regulation of biological process	80	6.0
	anatomical structure formation	71	5.4
	negative regulation of biological process	66	5.0
	immune system process	53	4.0
	multi-organism process	22	1.7
	growth	18	1.4
	biological adhesion	14	1.1
	locomotion	14	1.1
	reproduction	14	1.1
	reproductive process	14	1.1
	viral reproduction	4	0.3
**Cellular Component**	cell part	1084	81.9
	cell	1084	81.9
	organelle	733	55.4
	macromolecular complex	402	30.4
	organelle part	384	29.0
	membrane-enclosed lumen	140	10.6
	envelope	80	6.0
	extracellular region	32	2.4
	extracellular region part	12	0.9
	synapse	6	0.5
	synapse part	4	0.3
**Molecular Function**	binding	770	58.2
	catalytic activity	440	33.3
	structural molecule activity	77	5.8
	transporter activity	70	5.3
	transcription regulator activity	46	3.5
	molecular transducer activity	46	3.5
	enzyme regulator activity	30	2.3
	translation regulator activity	29	2.2
	electron carrier activity	11	0.8
	antioxidant activity	5	0.4

**Table 3:** Unigenes annotated with the GO term immune system process

**Sequence name**	**Sequence description**	**Hit AC**	**Clustered EST**
Cluster1088	fucolectin	Q7SIC1	1
Cluster1225	endoplasmic reticulum aminopeptidase 1	Q9NZ08	1
Cluster1249	transcription factor sp2	Q02086	1
Cluster1357	complement c3	P98093	1
Cluster1410	b-cell lymphoma 6 protein homolog	P41183	1
Cluster1474	matrix metalloproteinase-9	P14780	1
Cluster1562	serine threonine-protein phosphatase subunit	P30153	1
Cluster1638	inosine-5 -monophosphate dehydrogenase 2	Q3SWY3	1
Cluster1667	chemokine-like factor	Q9UBR5	1
Cluster1692	60 kda heat shock mitochondrial	Q5ZL72	1
Cluster1821	transcription elongation factor	Q4KLL0	1
Cluster1865	serine threonine-protein kinase tbk1	Q9WUN2	1
Cluster1872	dedicator of cytokinesis protein 2	Q92608	1
Cluster1891	complement c3	P98093	1
Cluster1908	interferon regulatory factor 4	Q64287	1
Cluster2109	sh2 domain-containing protein 1a	B2RZ59	1
Cluster2173	bisphosphate phosphodiesterase gamma-2	Q8CIH5	1
Cluster2189	ig heavy chain v-iii region cam	P01768	2
Cluster2214	complement c3	P12387	2
Cluster2253	calreticulin	P18418	2
Cluster2255	ap-2 complex subunit sigma-1	P62744	2
Cluster2335	myosin-if	O00160	2
Cluster2337	adenylate kinase mitochondrial	Q1L8L9	2
Cluster2342	40s ribosomal protein s14	P62263	2
Cluster2345	apoptotic chromatin condensation inducer	Q9UKV3	2
Cluster244	MHC I-related gene protein	Q95460	1
Cluster2440	ubiquitin thioesterase otub1	Q96FW1	2
Cluster2466	nf-kappa-b inhibitor alpha	P25963	2
Cluster2474	toll-interacting protein	A2RUW1	2
Cluster2602	moesin	P26038	3
Cluster2612	beta-2-microglobulin	Q04475	3
Cluster265	myosin-9	P14105	1
Cluster2659	proteasome maturation protein	Q3SZV5	3
Cluster2663	apoptotic chromatin condensation inducer	Q9UKV3	3
Cluster2706	cd81 antigen	P35762	3
Cluster2717	complement -binding mitochondrial	Q07021	4
Cluster2828	integrin alpha-l	P24063	6
Cluster2869	moesin	P26038	8
Cluster2872	beta-2-microglobulin	O42197	8
Cluster2877	c-x-c chemokine receptor type 4	P61072	8
Cluster2908	fucolectin	Q7SIC1	12
Cluster311	proteasome subunit beta type-9	Q8UW64	1
Cluster33	inosine-5 -monophosphate dehydrogenase 1	P20839	1
Cluster490	paired box protein pax-5	Q02548	1
Cluster493	nucleosome assembly protein 1-like 1-a	Q4U0Y4	1
Cluster588	cysteine-rich protein 2	Q9DCT8	1
Cluster634	interleukin enhancer-binding factor 2 homolog	Q6NZ06	1
Cluster668	zinc finger e-box-binding homeobox 1	P36197	1
Cluster780	kinase catalytic subunit delta isoform	O35904	1
Cluster789	cd81 antigen	P35762	1
Cluster812	interferon regulatory factor 1	P15314	1
Cluster937	high mobility group protein b3	Q32L31	1
Cluster999	aminoacyl trna synthetase protein	Q12904	1

**Table 4:** Unigenes annotated with the GO term response to virus

**Sequence name**	**Sequence description**	**Hit AC**	**Clustered EST**
Cluster2255	ap-2 complex subunit sigma-1	P62744	2
Cluster2287	interferon-induced gtp-binding protein	Q8JH68	2
Cluster2379	40s ribosomal protein s15a	P62244	2
Cluster2877	c-x-c chemokine receptor type 4	P61072	8

**Table 5:** Unigenes annotated with the GO term response to bacterium

**Sequence name**	**Sequence description**	**Hit AC**	**Clustered EST**
Cluster12	histone h2a	P02264	1
Cluster1225	endoplasmic reticulum aminopeptidase 1	Q9NZ08	1
Cluster1269	lysozyme c	P85045	1
Cluster1910	akirin-2	Q25C79	1
Cluster2173	phosphatidylinositol phosphodiesterase gamma-2	Q8CIH5	1
Cluster2335	myosin-if	O00160	2
Cluster2543	histone h1	P06350	2
Cluster2861	histone h1	P06350	7
Cluster566	histone h1	P06350	1

### KEGG pathway analysis

A total of 989 of the 3,027 were assigned a KEGG ontology (KO) annotation; they were mapped to 201 KEGG pathways. Three most frequently occurring KEGG pathways were ribosome, oxidative phosphorylation, and proteasome. 68 unigenes mapped to immune-related pathways including leukocyte transendothelial migration, antigen processing and presentation, chemokine signalling pathway, and T cell receptor signalling pathway (Table 6). We found that 28 unigenes from head kidney in grass carp have been reported to be involved in the following pathways, Toll-like receptor signalling pathway, RIG-I-like receptor signalling pathway and the NOD-like receptor signalling pathway (Table 7).

**Table 6:** The most represented KEGG pathways in the unigene data set

**Pathway**	**Mapping genes**	**Categories**
Ribosome	60	Genetic Information Processing
Oxidative phosphorylation	53	Metabolism
Proteasome	32	Genetic Information Processing
Spliceosome	31	Genetic Information Processing
Lysosome	28	Cellular Processes
Purine metabolism	25	Metabolism
Endocytosis	24	Cellular Processes
Regulation of actin cytoskeleton	24	Cellular Processes
Cell cycle	19	Cellular Processes
Leukocyte transendothelial migration	18	Organismal Systems
Pyrimidine metabolism	17	Metabolism
MAPK signalling pathway	17	Environmental Information Processing
Antigen processing and presentation	17	Organismal Systems
Chemokine signalling pathway	17	Organismal Systems
Tight junction	16	Cellular Processes
T cell receptor signalling pathway	16	Organismal Systems

**Table 7:** Mapping genes in fish primary non-specific immune pathways

**Pathway**	**Mapping genes**	**Containing ESTs**
Toll-like receptor signalling pathway	8	16
RIG-I-like receptor signalling pathway	11	20
NOD-like receptor signalling pathway	9	17

### Expression profiling analysis

By Solexa sequencing, we obtained 7,696,804 and 6,136,889 raw tags from the transcriptomes of head kidney tissue from grass carp before and after GCRV infection, respectively. After removing low quality sequences, adapter sequences and single copy sequence the cleaned tag numbers were 7,188,005 and 5,724,526, respectively. The final numbers of non-redundant distinct tags were 152,826 and 105,653 before and after GCRV infection, respectively. All tags were submitted to SRA at NCBI under the accession no. SRA052520.2. Of the distinct tags, 22,144 were differentially expressed by more than 2-fold between the GCRV-infected and uninfected groups.

These 22,144 differentially expressed tags mapped to 3,027 unigenes using SeqMap [30]. Of the differentially expressed tags, 679 (3.1%) mapped to 483 differentially expressed unigenes (16.0%); 145 of the unigenes were up-regulated genes, 307 were down-regulated genes. The remaining 31 unigenes mapped to tags that exhibited both up and down regulation, and so these unigenes were not included in the statistics. The up- and down-regulated genes were mainly annotated with the GO terms, genetic information processing, metabolism, and cellular processes and 16 unigenes were annotated with the GO term immune-related (Table 8). We found 54 tags that mapped onto 42 of the 221 unknown unigenes. These are potentially infection related novel genes; 15 of them were up-regulated between the GCRV-infected and uninfected groups, and 27 were down-regulated genes (Table 9).

**Table 8:** Differentially expressed unigenes annotated as immune-related

**Sequence name**	**Description**	**log2Ratio (VP/CP)**	**Up-Down**
cichka_Cluster2189.seq. Contig1	ig heavy chain v-iii region cam	9.552669098	Up
cichka_Cluster2214.seq. Contig1	complement c3	−1.234417227	Down
cichka_Cluster2335.seq. Contig1	myosin-if	−1.616395009	Down
cichka_Cluster2337.seq. Contig1	adenylate kinase mitochondrial	−2.622261042	Down
cichka_Cluster2612.seq. Contig1	beta-2-microglobulin	14.96510786	Up
cichka_Cluster2717.seq. Contig1	complement -binding mitochondrial	2.831849484	Up
cichka_Cluster2828.seq. Contig1	integrin alpha-l	−3.476196501	Down
cichka_Cluster2872.seq. Contig1	beta-2-microglobulin	−2.257387843	Down
cichka_Cluster2877.seq. Contig1	c-x-c chemokine receptor type 4	−2.941536738	Down
cichka_Cluster2908.seq. Contig1	fucolectin	−3.57091306	Down
cichka_Cluster2379.seq. Contig1	40s ribosomal protein s15a	−2.133495724	Down
cichka_Cluster1269	lysozyme c	−5.60930435	Down
cichka_Cluster634	interleukin enhancer-binding factor 2 homolog	−8.383704292	Down
cichka_Cluster812	interferon regulatory factor 1	2.652601218	Up
cichka_Cluster1474	matrix metalloproteinase-9	−5.851050959	Down
cichka_Cluster1667	chemokine-like factor	−8.189824559	Down

**Table 9:** Potentially novel differentially expressed unigenes

**Sequence name**	**log2 Ratio(VP/CP)**	**Up-Down**
cichka_Cluster1	−1.30897451703681	Down
cichka_Cluster1004	−8.18982455888002	Down
cichka_Cluster1074	−4.68008319087111	Down
cichka_Cluster1080	1.5772610962369	Up
cichka_Cluster1095	1.70760741456741	Up
cichka_Cluster1139	−3.01282922395069	Down
cichka_Cluster1321	−1.17687776208408	Down
cichka_Cluster1418	2.57740490960702	Up
cichka_Cluster144	−2.37056287013824	Down
cichka_Cluster1502	−2.69938241135805	Down
cichka_Cluster153	9.00842862207058	Up
cichka_Cluster155	−4.35320513151951	Down
cichka_Cluster1567	−3.23219204494701	Down
cichka_Cluster1689	−1.24599865006401	Down
cichka_Cluster18	−8.55458885167764	Down
cichka_Cluster1830	5.67155018571725	Up
cichka_Cluster1847	−1.8154025874359	Down
cichka_Cluster19	−7.4998458870832	Down
cichka_Cluster1931	1.44222232860508	Up
cichka_Cluster2016	2.84923580318831	Up
cichka_Cluster2063	−3.29278174922784	Down
cichka_Cluster219	8.44708322620965	Up
cichka_Cluster2432.seq. Contig1	−1.12271915825313	Down
cichka_Cluster2506.seq. Contig1	−2.26096007759593	Down
cichka_Cluster2646.seq. Contig1	−8.18982455888002	Down
cichka_Cluster2651.seq. Contig1	−8.32192809488736	Down
cichka_Cluster2765.seq. Contig1	−8.38370429247405	Down
cichka_Cluster291	3.07771266869725	Up
cichka_Cluster2966.seq. Contig1	−5.46317402032312	Down
cichka_Cluster317	−2.29418310440446	Down
cichka_Cluster357	3.48529281620541	Up
cichka_Cluster468	−1.41853954357293	Down
cichka_Cluster482	1.43096228428556	Up
cichka_Cluster559	7.82654848729092	Up
cichka_Cluster613	−1.71345884128158	Down
cichka_Cluster619	−3.62148837674627	Down
cichka_Cluster625	1.46068016483455	Up
cichka_Cluster751	1.83711846346595	Up
cichka_Cluster788	1.13154390971446	Up
cichka_Cluster790	−5.47619650111671	Down
cichka_Cluster837	−2.25442127552909	Down
cichka_Cluster891	−1.33599920243744	Down

### Cloning and expression regulation analysis of the novel genes

**Figure 2:**
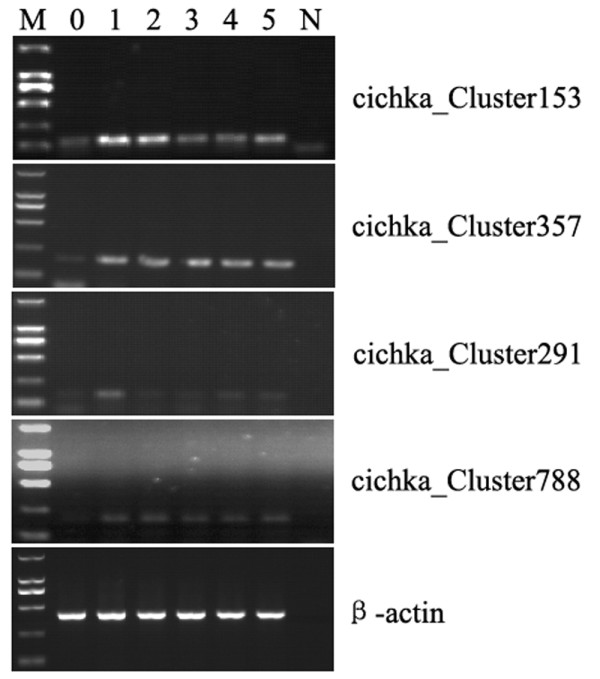
**RT-PCR verification of the novel infection-related genes.** M, maker; 0, non-infected tissue; 1, 1 day after infection; 2, 2 days after infection; 3, 3 days after infection; 4, 4 days after infection;5, 5 days after infection; N, the negative control.

Using semi-quantitative RT-PCR, we examined the gene expression changes of the 42 potentially novel unigenes that were detected in the head kidney after viral infection. By comparing the 1, 2, 3, 4, and 5 day post-infection samples and the samples from the control group, we found four unigenes that showed a significant response to the viral infection: cichka_Cluster153 and cichka_Cluster291 were up-regulated in days 1 and 2 post-infection after which their expressions returned to the starting level; cichka_Cluster357 and cichka_Cluster788 were up-regulated in days 1 and 2 post-infection, and the increased expression levels were maintained till day 5 (Figure 2).

The full-length cDNA sequences of these four unigenes were 2,057 bp (cichka_Cluster291, JQ412736), 2,288 bp (cichka_Cluster357, JQ412737), 1,044 bp (cichka_Cluster788, JQ412738) and 1,387 bp (cichka_Cluster153, JQ412739) encoding polypeptides of 586, 322, 142 and 155 amino acids, respectively. BLAST searches revealed that cichka_Cluster291 can encode a protein that is similar to the vertebrate endonuclease domain containing protein, cichka_Cluster357 can encode a protein that is similar to the vertebrate ankyrin repeat domain 10 protein, cichka_Cluster788 can encode a protein that is similar to the CST complex subunit TEN1; for the cichka_Cluster153 encoded protein, no similar sequences were found in the databases that we searched, suggesting that cichka_Cluster153 may represent a novel gene in grass carp. We used the SMART server [31] to predict the domain structure of the 42 novel unigenes and found that 83.02% of them contained the endonuclease domain 1 that is found in proteins that are involved in the apoptosis pathway, and 35.22% contained the ankyrin repeat domain that is present in proteins that are involved in pathways that include the B cell receptor signalling pathway, the T cell receptor signalling pathway, and the apoptosis pathway. The cichka_Cluster788 unigene contained no obvious structural domains; the cichka_Cluster153 encoded protein contained two transmembrane domains and may be a transmembrane protein.

## Discussion

Currently, there are about 6,915 sequences of grass carp in the public databases. This situation does not reflect the extremely important breeding position of grass carp. In this study, we built a head kidney non-normalized cDNA library of healthy grass carp and obtained 3,027 unigene EST sequences. This library greatly enriches the available genomic data for grass carp and lays an important foundation for the discovery of novel genes and for their functional investigation.

GO analysis revealed that the annotated unigenes were mainly related to genes involved in basic biological processes such as cellular process (25.5%), metabolic process (19.1%) and biological regulation (10.8%). This functional distribution is similar to the EST distributions reported earlier in the head kidney of zebrafish [32] and sea bass [10].

Of the unigenes that were similar to immune-related genes, 66 unigenes were annotated as associated with the immune process; 53 were related to the immune system process, 4 were annotated as response to virus, and 9 were related to response to bacteria. Among the 989 unigenes that were assigned KO annotations, 68 were mapped to immune-related pathways that included leukocyte transendothelial migration, antigen processing and presentation, chemokine signalling pathway and T cell receptor signalling pathway. By examining the literature, we found that 28 of the unigenes in grass carp head kidney were related to fish genes that were reported to be involved in the Toll-like receptor signalling pathway, the RIG-I-like receptor signalling pathway and the NOD-like receptor signalling pathway. Clearly, head kidney tissue plays an important role in immune processes in fish. EST databases of head kidney tissue are likely to become important resources in which immune-related genes can be identified.

In the 3,027 unigene library of head kidney in grass carp, 7.3% (221) failed to match any of the sequences in the three public databases that were searched. Of the 10 unigenes that were the most highly expressed in grass carp head kidney, 9 were unknown sequences (Table 10). This could be partly because sequence data for fish is still very scarce, and partly because fish head kidney tissue may contain tissue-specific or species-specific genes. EST databases can be important resources for identifying unknown genes in fish [33-35]. In recent years, the fish transcriptome has been used to study the regulation of gene expression. Pardo et al (2008) conducted a comparative study of turbot expression profiles in the main immune tissue before and after pathogen infection to find genes that were related to immune response and disease resistance [36]. Chini et al (2008) carried out a comparative study of reproductive development-related tissues in bluefin tuna using transcriptome research methods to explore the molecular mechanism of gonadal development and maturity split [13]. Indeed, comparative transcriptome analysis can be used, not only to investigate the mechanisms of expression and regulation of known genes, but also as an effective means to find important and novel function-related genes.

**Table 10:** Ten most highly expressed unigenes in the head kidney of healthy grass carp

**Sequence name**	**ORF length**	**Clustered ESTs**	**Description**
Cluster2971	159	1114	Unknown
Cluster2970	267	251	hybrid granulin
Cluster2969	132	166	hypothetical 18 K protein
Cluster2968	282	109	Unknown
Cluster2967	273	123	Unknown
Cluster2966	267	78	hypothetical protein
Cluster2965	132	85	Unknown
Cluster2964	0	83	Unknown
Cluster2963	279	63	Unknown
Cluster2962	108	55	Unknown

## Conclusion

We carried out a comparative analysis to find differences in the Solexa expression profiles of head kidney in grass carp before and after infection, and identified 42 unigenes of unknown function that showed differential expression in response to the pathogen. After RT-PCR validation of the cDNA and gene structure analysis, we found a potentially novel immune-related gene. Based on its response to viral infection and the prediction that it might encode a membrane protein, we speculate that this novel gene may encode a virus receptor or a protein that mediates the immune signalling pathway at the cell surface. We intend to further investigate the function of this gene in a future study. Our findings confirm that fish tissue-specific EST databases combined with comparative transcriptome analysis are effective tools that can direct the discovery of novel functional genes.

## Competing interests

The authors declare that they have no competing interests.

## Authors’ contributions

CJ carried out the experiments and drafted the manuscript. LC and DFK conducted the database searches and bioinformatics analysis. HR and ZZY participated in the study design and in the manuscript preparation. LLJ was involved in the experiments. WYP was overall responsible for the project and finalized the manuscript. All authors read and approved the final manuscript.
